# A Rare Case of Clear Cell Carcinoma, Müllerian Type in the Renal Pelvis of a 21-Year-Old Woman

**DOI:** 10.1155/2018/1521598

**Published:** 2018-04-01

**Authors:** Diandra Perez, Rana Naous

**Affiliations:** Department of Pathology, SUNY Upstate Medical University, Syracuse, NY 13210, USA

## Abstract

Clear Cell Carcinomas of Müllerian origin are extremely rare within the upper urinary system. Their morphology is identical to that of the Clear Cell Carcinomas of the female genital tract. When they arise in the urinary tract, it is thought to be due to ectopic Müllerian embryogenesis. Here, we present a case of a 21-year-old woman with a Clear Cell Carcinoma, Müllerian type, arising from the renal pelvis. Histologically, it consisted of tubulopapillary architecture with associated foamy macrophages and a mucinous background. The neoplastic cells exhibited variably sized round nuclei with prominent nucleoli, eosinophilic to vacuolated cytoplasm with occasional intracytoplasmic mucin vacuoles, and a hobnail appearance. Immunohistochemical stains showed that the neoplastic cells were positive for Pax-8, p53, CK7, HMWK 903, and INI-1 and focally positive for p504s (AMACR). The neoplastic cells were negative for GATA-3, CK5/CK6, p63, CK20, and CDX-2 immunostains, ruling out urothelial or enteric phenotype. Additional immunostains performed by an outside institution showed that the neoplastic cells were positive for HNF-1*β*. The overall morphology and immunophenotype were consistent with Clear Cell Carcinoma of Müllerian origin arising from the renal pelvis. Follow-up revealed no metastasis or other tumor sites, supporting that this was the primary location.

## 1. Introduction 

Müllerian neoplasms of the urinary system are a rare entity of neoplasms that are subdivided into Clear Cell Carcinoma (CCC) and Endometrioid Carcinoma. There are several controversial theories supporting their pathogenesis, including remnants of embryological reproductive precursors within the urinary tract, endometriosis, and Müllerianosis. However, one thing is agreed upon, and that is their morphology, which shares a striking resemblance to their female genital tract counterpart. CCC, although rare itself, is more common than Endometrioid Carcinoma. It is usually seen in the bladder or urethra, more commonly in females, but a Clear Cell Carcinoma arising from the renal pelvis is exceedingly rare.

## 2. Case Description

**Figure 1:**
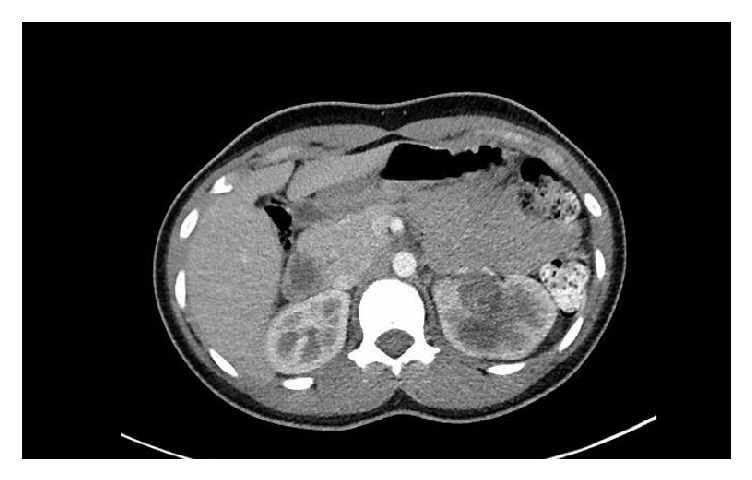
Abdominal computed tomography scan reveals a mass in the left pelvicalyceal system with associated moderate to severe hydronephrosis.

A 21-year-old African American female presented with a long history of left flank pain for which she had been evaluated multiple times without success. Cytopathology of voided urine showed atypical cytology composed of groups of urothelial cells with enlarged, hyperchromatic nuclei. Urinalysis was positive for microhematuria and hemoglobinuria. A CT scan revealed a 6 cm enhancing left renal mass within the collecting system and hydronephrosis (Figure 1). The patient had no prior history of Sickle Cell Disease/Trait or any history of other primary malignancies. No urethral diverticulum was found on physical exam or imaging.

The patient underwent laparoscopic radical nephrectomy. Pathologic examination of the specimen revealed a 5.5 × 5.5 × 1.0 endophytic, ill-defined, friable, and focally necrotic pale-yellow renal mass with a complex and full renal hilum containing three renal veins, one renal artery, and one ureter. The mass was attached by a pedicle arising from the renal pelvis wall with an additional attachment point that could not grossly exclude renal sinus involvement.

## 3. Histologic Findings

**Figure 2:**
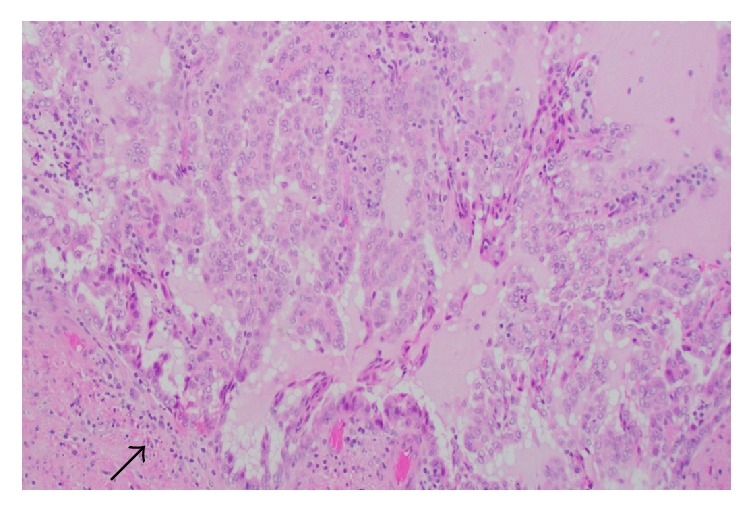
Tumor appears to be arising from the renal pelvis (arrow) and has a papillary, micropapillary, and tubular architecture (200x, H&E stain).

**Figure 3:**
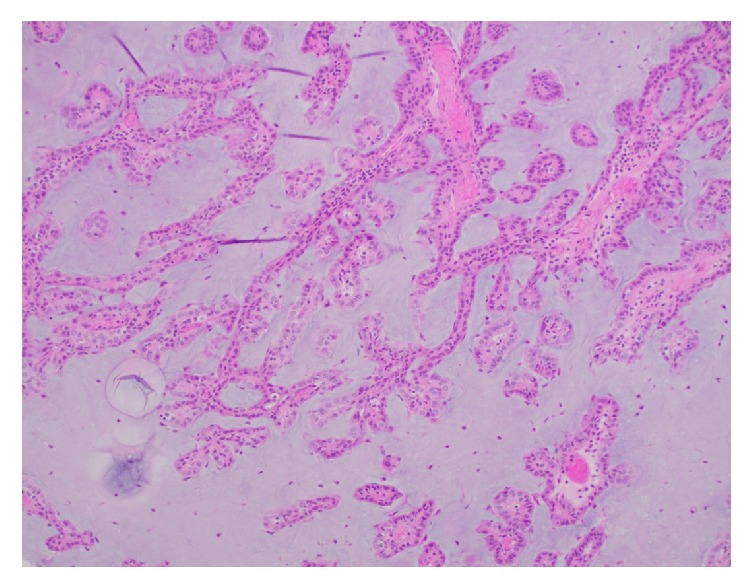
Papillary tumor fronds floating within a mucinous background (200x, H&E stain).

**Figure 4:**
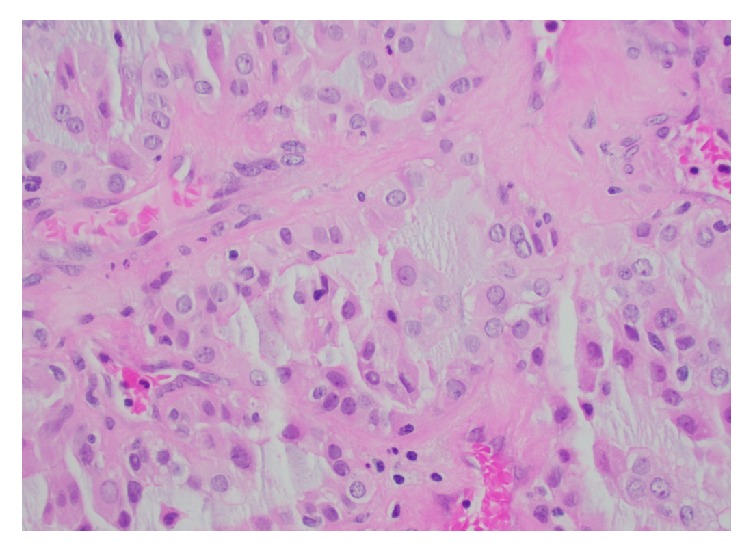
Tumor cells have occasionally prominent nucleoli and eosinophilic to vacuolated cytoplasm (400x, H&E stain).

Microscopic examination revealed a 5.5 cm neoplasm located in the renal pelvis arranged in a papillary, micropapillary, and tubular architecture (Figure 2) with associated foamy macrophages and mucinous background (Figure 3). The neoplastic cells had variably sized round nuclei with prominent nucleoli, eosinophilic to vacuolated cytoplasm with occasional intracytoplasmic mucin vacuoles, and a hobnail appearance (Figure 4). The tumor also exhibited a cystic arrangement in variable sizes and shapes. Necrosis was noted in 5–10% of the neoplasm; however, mitotic figures were scarce. Additionally, the neoplasm seemed to reside entirely within the renal pelvis with focal involvement of the renal medulla, without evidence of extension into the renal pelvic subepithelial connective tissue or peripelvic fat. The vascular and ureteral margins were negative and there was no evidence of lymphovascular invasion.

**Figure 5:**
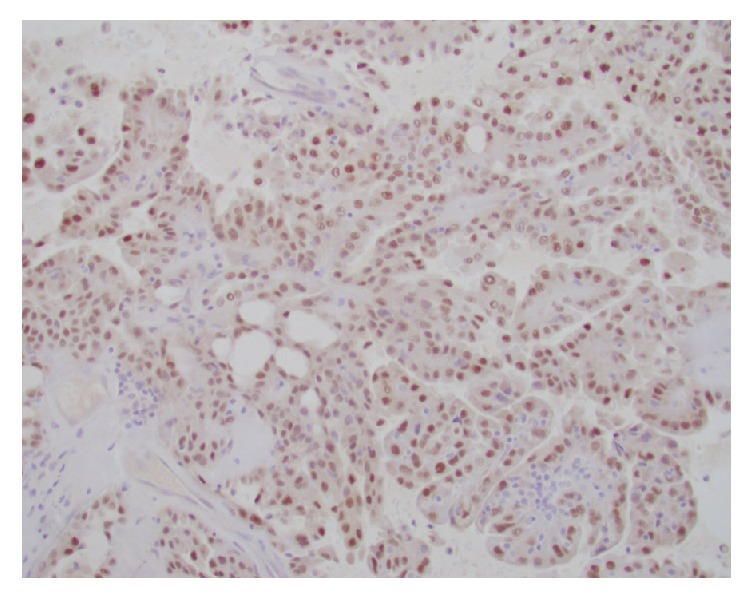
Diffuse nuclear positivity to Pax-8 immunostain.

**Figure 6:**
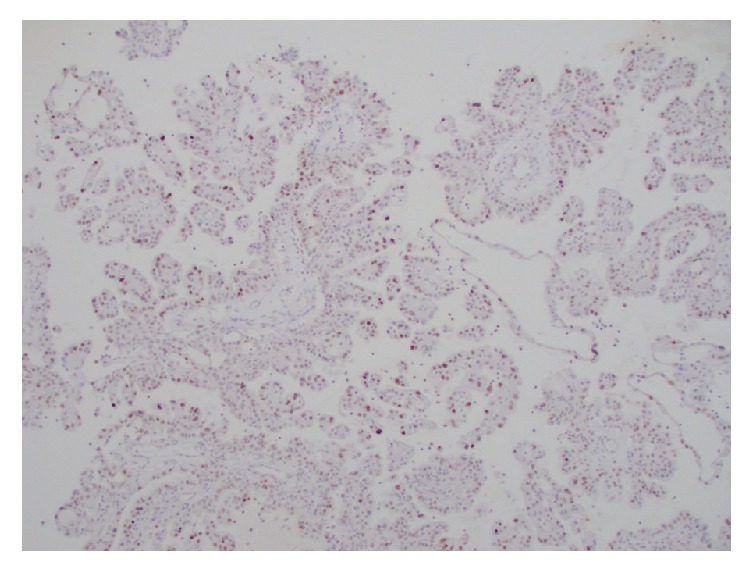
Diffuse nuclear positivity to p53 immunostain.

**Figure 7:**
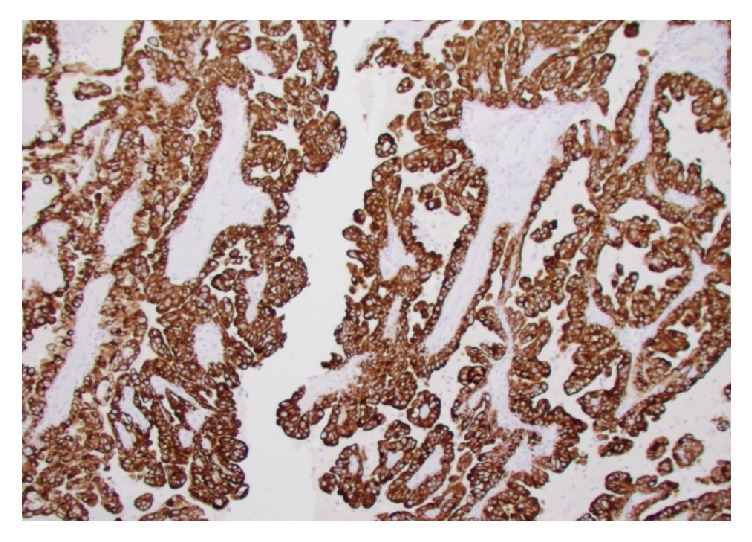
Diffuse membranous positivity to CK7 immunostain.

**Figure 8:**
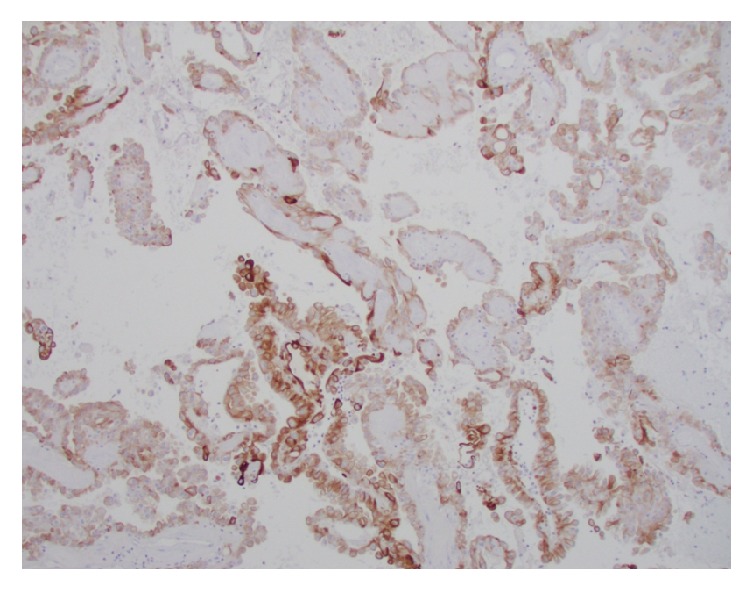
Diffuse membranous positivity to HMWK 903 immunostain.

**Figure 9:**
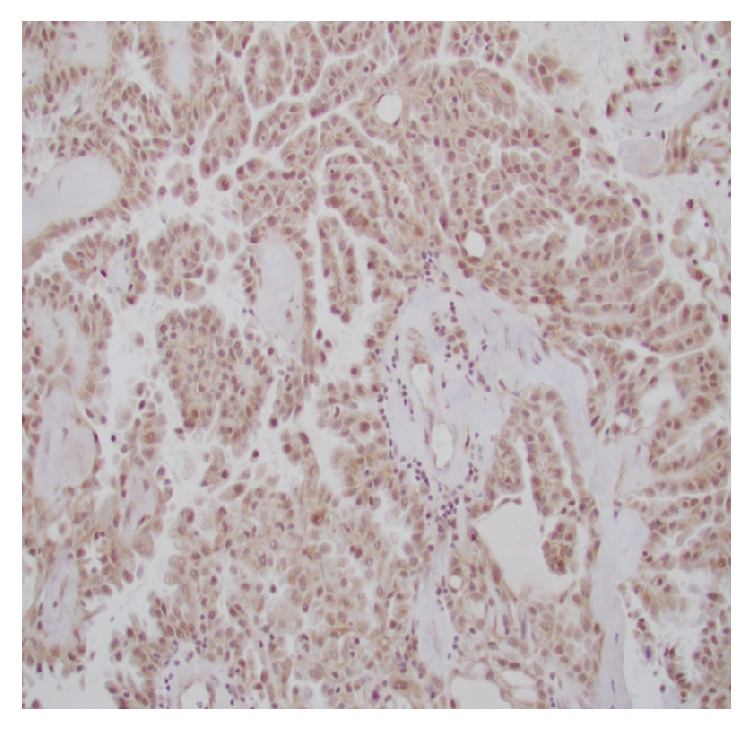
Diffuse nuclear positivity to INI-1 immunostain.

**Figure 10:**
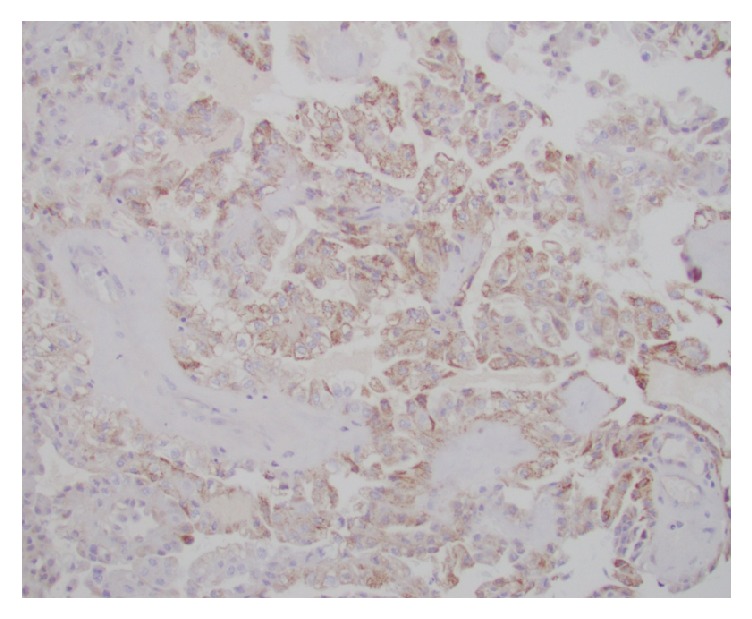
P504S (AMACR) immunostain was focally positive in the tumor cells.

**Figure 11:**
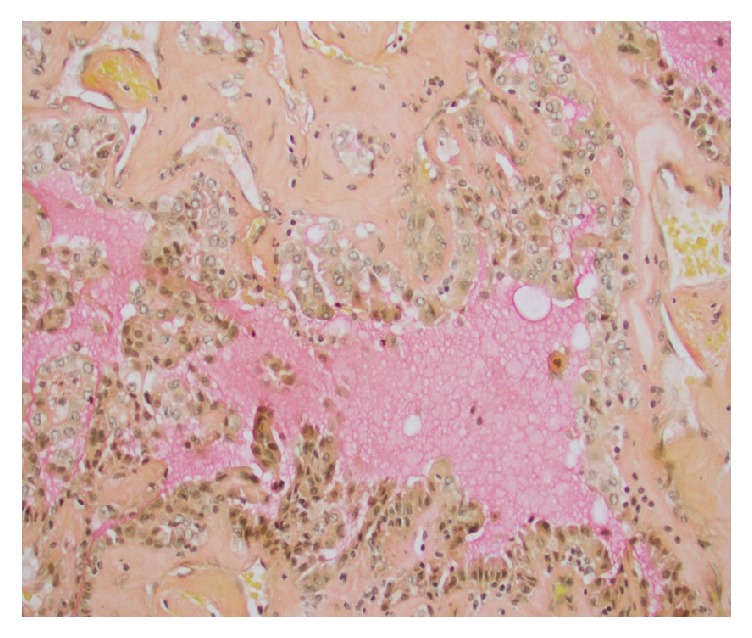
Mucicarmine stain highlighting the background mucin.

Immunohistochemical stains showed that the neoplastic cells were positive for Pax-8 (Figure 5), p53 (Figure 6), CK7 (Figure 7), HMWK 903 (Figure 8), and INI-1 (Figure 9) and focally positive for p504s (AMACR) (Figure 10). The neoplastic cells were negative for GATA-3, CK5/CK6, p63, CK20, and CDX-2 immunostains ruling out urothelial or enteric phenotype. ALK immunostain was performed to rule out an ALK rearrangement-associated Renal Cell Carcinoma and was negative. Mucicarmine stain (Figure 11) highlighted the background mucin as well as the intraluminal and intracytoplasmic mucin.

At this point, the case was sent out for an expert consultation at an outside institution where additional immunohistochemistry was performed. These showed that the tumor cells were positive for HNF-1*β* (pictures were not provided) and negative for ER, PR, napsin-A, TFE3, cathepsin-K, Fumarate hydratase, Succinate Dehydrogenase-B, MSH6, and PMS-2 immunostains. Additional sections of uninvolved renal parenchyma were taken to rule out endometriosis as a precursor and were negative.

Finally, it was concluded by the expert consultant that, based on this morphology and immunophenotype, the overall findings support the diagnosis of Clear Cell Carcinoma, Müllerian type arising from the renal pelvis.

## 4. Discussion

Clear Cell Carcinoma (CCC) is most commonly associated with the neoplasms of the female genital tract, some of which are famously associated with in utero exposure to diethylstilbestrol (DES) [1]. However, CCC can rarely be seen arising from the urinary tract. The classification of these neoplasms has undergone several changes over the years; they were previously classified by the WHO as Clear Cell Adenocarcinoma; the most recent WHO classification is Clear Cell Carcinoma, Müllerian type. Originally, these neoplasms were referred to as “mesonephric carcinoma,” since they were thought to arise from mesonephric ducts [2]. Although histologically identical to CCC of the female genital tract, the histogenesis of CCC of the urinary tract is not unanimously agreed upon. These neoplasms have been associated with endometriosis and Müllerian duct remnants [3, 4]. The Müllerian ducts, also called the paramesonephric ducts, are derived from the mesoderm in embryos of both genders but only go on to develop the reproductive organs in females and degenerate in males. In females, the Müllerian ducts give rise to the uterus, fallopian tubes, and the upper third of the vagina. During embryogenesis, these precursor cells can become displaced during migration and a possible nidus for endometriosis [5].

Although rare in general, CCC of the lower urinary tract is more common in the bladder and urethra, with a female predominance. Cases in males are even rarer still but have also been noted, such as a case of CCC of the prostate [6]. CCC of the upper urinary tract is extremely rare, with only 3 cases reported to the best of our knowledge. One case belongs to a 73-year-old woman with a CA125-producing Clear Cell Adenocarcinoma of the upper ureter and renal pelvis that metastasized to the lungs, unfortunately resulting in her death [7], a second case of a 43-year-old woman with a HNF-1*β* positive Clear Cell Adenocarcinoma of the renal pelvis [8], and a third unique case of Clear Cell Adenocarcinoma of the renal pelvis in a 54-year-old male [9].

In this case, given that the tumor arose from the superficial medulla and renal pelvis, the differential diagnosis included renal epithelial tumors of the Renal Cell Carcinoma (RCC) family such as Papillary RCC, Hereditary Leiomyomatosis and Renal Cell Carcinoma-associated RCC, ALK rearrangement-associated RCC, and Succinate Dehydrogenase-Deficient RCC.

Papillary Renal Cell Carcinoma (PRCC) is a malignant renal epithelial tumor that comprises approximately 10% of RCCs. PRCC is derived from the renal tubular epithelium and occurs in the renal cortex [2, 10]. It was considered in our differential diagnosis due to its characteristic papillary and tubular architecture. It is usually positive for Cytokeratin 7 and P504S (AMACR) immunostains which were also positive in our case; however, due to the location of the tumor, the diagnosis of Papillary RCC was considered unlikely.

Hereditary Leiomyomatosis and Renal Cell Carcinoma-associated Renal Cell Carcinoma is a rare subtype of the familial RCCs that follows an autosomal dominant pattern of germline fumarate hydratase mutations and usually affects the renal cortex and medulla [2, 11, 12]. It was also considered in our differential diagnosis due to its morphology that consists of a papillary or infiltrative growth pattern of large cells with abundant eosinophilic cytoplasm, large nuclei, and prominent inclusion-like eosinophilic nucleoli with perinucleolar clearing. In our case, the patient had no family history of any neoplasms and the fumarate hydratase immunostain was negative, making this diagnosis unlikely.

ALK rearrangement-associated Renal Cell Carcinomas are extremely rare entities, with less than ten cases reported in the literature, which tend to occur in pediatric patients with sickle cell trait. These neoplasms arise in the renal medulla and microscopically can have tubulopapillary architecture with large polygonal cells, eosinophilic cytoplasm, and intracytoplasmic lumina [2, 13, 14]. In our case, ALK immunostain was performed and was negative, ruling out this entity.

Succinate Dehydrogenase-Deficient Renal Cell Carcinomas are epithelial tumors arising from the renal parenchyma and account for up to 0.2% of all RCCs. The morphology consists of a solid, nested, or tubular pattern of cells with a vacuolated eosinophilic to clear cytoplasm. The succinate dehydratase genes (SDH) are usually lost in a double-hit inactivation, resulting in a loss of SDHB immunohistochemical staining [2, 15]. In our case, SDHB immunostain was performed and was negative; that is, there was no loss of the SDHB gene, arguing against this diagnosis.

In our case, the overall histology and immunophenotype fit best with the diagnosis of Clear Cell Carcinoma, Müllerian type. It is extremely rare to have Clear Cell Carcinoma at this location. However, in the absence of a primary neoplasm elsewhere and given the fact that Müllerian Rests or endometriosis was not identified, it is likely that the tumor arose within the pelvic urothelium.

Clear Cell Carcinomas can metastasize to lymph nodes and distant organs, but the overall prognosis is not well studied at this time due to rarity of cases and lack of long-term follow-up. In this case, the patient returned for follow-up care to receive a PET scan, pelvic ultrasound, and Lynch Syndrome Study. All studies were negative, supporting nonmetastatic primary disease within the renal pelvis and a possibly favorable outcome.

## 5. Conclusion

In summary, we described a rare case of Clear Cell Carcinoma of Müllerian origin arising from the renal pelvis in a 21-year-old female. To the best of our knowledge, this is the fourth case of CCC of the upper urinary tract. Due to the rarity of these cases and lack of long-term follow-up, the prognosis of such an entity remains unclear.
